# Sustainable Alkali-Activated Self-Compacting Concrete for Precast Textile-Reinforced Concrete: Experimental–Statistical Modeling Approach

**DOI:** 10.3390/ma17246280

**Published:** 2024-12-22

**Authors:** Vitalii Kryzhanovskyi, Jeanette Orlowsky

**Affiliations:** Faculty of Architecture and Civil Engineering, TU Dortmund University, 44227 Dortmund, Germany; jeanette.orlowsky@tu-dortmund.de

**Keywords:** self-compacting concrete, fine grade concrete, brick powder, recycling, alkaline activator, experimental–statistical modeling, optimization, CO_2_ emission

## Abstract

Industrial and construction wastes make up about half of all world wastes. In order to reduce their negative impact on the environment, it is possible to use part of them for concrete production. Using experimental–statistical modeling techniques, the combined effect of brick powder, recycling sand, and alkaline activator on fresh and hardened properties of self-compacting concrete for the production of textile-reinforced concrete was investigated. Experimental data on flowability, passing ability, spreading speed, segregation resistance, air content, and density of fresh mixtures were obtained. The standard passing ability tests were modified using a textile mesh to maximize the approximation to the real conditions of textile concrete production. To determine the dynamics of concrete strength development, compression and flexural tests at the ages of 1, 3, 7, and 28 days and splitting tensile strength tests of 28 days were conducted. The preparation technology of the investigated modified mixtures depending on the composition is presented. The resulting mathematical models allow for the optimization of concrete compositions for partial replacement of slag cement with brick powder (up to 30%), and natural sand with recycled sand (up to 100%) with the addition of an alkaline activator in the range of 0.5–1% of the cement content. This allows us to obtain sustainable, alkali-activated high-strength self-compacting recycling concrete, which significantly reduces the negative impact on the environment and promotes the development of a circular economy in the construction industry.

## 1. Introduction

The increased rate of industrialization makes it necessary to pay special attention to the recycling of industrial and construction wastes. This approach favors the development of a sustainable construction ecosystem, which helps prevent negative environmental impacts, in particular, reducing CO_2_ emissions. From the point of view of the circular economy and existing knowledge in the field of concrete and cement technology, alternative binders of man-made origin are of particular interest [1,2,3]. These materials have so-called hidden hydraulic (pozzolanic) properties. The peculiarity of their chemical composition is the increased content of amorphous silicon dioxide (SiO_2_), aluminum oxide (Al_2_O_3_) and iron oxide (Fe_2_O_3_) of not less than 70%, which, interacting with free lime Ca(OH)_2_ during cement hydration, forms additional hydrate C-S-H phases, which provides an increase in the mechanical properties of cement concrete [4,5,6]. The most widespread pozzolanic materials of anthropogenic origin are microsilica, fly ash, and blast furnace granulated slag.

As a result of the closure of the coal industry in Germany in 2018, such pozzolanic material as fly ash has become scarce. The declining trend of fly ash utilization in cement and ready-mix concrete production started from 1999 to 2009. During this period, the application of fly ash declined by 36.9%. According to the data [7], the remaining fly ash stockpile in Germany in 2020 was 1.3 million tones, which will be used up by the end of 2025 [8]. At the same time, due to increasing energy costs [9], there is a tendency to reduce smelting operations. For example, steel production in 2023 decreased by 4% and electric arc furnace production by 20% [10]. In turn, this may lead to a decline in producing an active mineral additives such as microsilica. Blast furnace granulated slag is relatively available at the moment, but the shortage of fly ash and microsilica on the local German market requires the search for new sources of supplementary cementitious materials. For this reason, industrial waste such as brick powder (BP) is of interest. According to [11], there are about 80 members of this industry in Germany who are potential producers of BP, which can be used as a partial replacement for cement in concrete production. At present, there is no unified recommendation for the use of this industrial waste in the concrete industry, but several studies have noted its effectiveness.

### 1.1. BP for Cement Replacement in Concrete Production

In [12], it was reported that up to 15% of pozzolanic cement can be replaced with BP for the production of C20/25 concrete. The maximum grain size of the BP used did not exceed 75 µm. The experiment in [13] demonstrates the effectiveness of substituting ordinary Portland cement with up to 25% BP with a grain size not exceeding 60 µm. However, it is important to maintain a W/C ratio of 0.26 and a sand ratio of 33%. Concretes with compressive strength of at least 50 MPa and flexural strength of 10–12 MPa were obtained. The authors of [14] achieved concrete of class C16/20 on ordinary cement at partial replacement with BP 5%. Further addition of BP negatively affected the compressive and splitting tensile strength but increased the flexural strength by 5% when 10% BP was used. It has been observed that the workability of concrete mixtures decreases by more than 35% when only 5% BP is introduced. The authors of [15] demonstrated the positive effect of replacing 10% of ordinary cement with BP and the design concrete compressive strength was 34.2 MPa, higher compared to the control one of 30.8 MPa; in addition, the splitting tensile strength was also higher—3.4 MPa for the modified concrete and 3.1 MPa for the control. In turn, [16] also confirmed the effectiveness of using 10% BP with a maximum grain size not exceeding 75 µm. The compressive, flexural, and splitting tensile strength were greater than the control specimen’s by 9.7%, 11.8%, and 1.25%, respectively. Similar results are reported in [17]. The workability of the concrete mixtures decreased to 6.1 cm, while the control composition had a workability of 12 cm. The equal effect of exposure of 10% and 20% BP with a grain size of 90 µm was shown in [18]. The compressive, flexural, and splitting tensile strength were higher than the base mixture’s by 9.4%, 5%, and 11.1%, respectively. The self-compacting concrete (SCC) was prepared using 1–5% BP by weight of cement. The maximum flowability (SF) of the fresh mixture was 688 mm at 2.5% and 5% BP, and the maximum compressive strength at 28 days of 50 MPa was achieved at 1% BP content [19]. In the study of cement–sand mortars, it was determined that BP can effectively substitute cement in an amount of 15%. Thus, the compressive and flexural strength was 1.6 times higher than that of control compositions [20]. In the case of BP application in the range of 5–50% for ultra-high-performance concrete (UHPC), the authors found that, in all cases, the strength values decreased; however, the possibility of obtaining UHPC with compressive strength not lower than 120 MPa and flexural strength not lower than 17 MPa in 28 days of hardening is emphasized [21].

### 1.2. Recycling Aggregates for Concrete Application

Apart from the need to improve concrete production technology by using supplementary cementitious materials to control CO_2_ emissions and reduce the energy consumption of cement plants, there is another challenge in ensuring concrete quality, namely, the use of recycled concrete aggregates. According to [22,23], the trend of concrete demolition waste (CDW) continues to increase, and, already by 2025, the annual global volume will increase to 2.2 billion tones. Consequently, a proactive approach to the reuse of CDW, e.g., as aggregates for concrete production, as described in the programs detailed in [24], is already necessary.

German standards [25,26,27] regulate the possibility of using recycled aggregates with a coarseness of more than 4 mm for concrete production. Individual papers describe the concept and technology of reusing CDW for sustainable concrete and building material technology [28,29,30,31].

Among the negative aspects of using CDW as an aggregate, it is important to note that it can lead to a drop in compressive strength when the quantity of recycling and the W/C ratio increases [32,33,34,35]. The flexural strength decreases with an increasing amount of recycling, but this decrease is less pronounced compared to that of the compressive strength [36,37,38]. At the same time, the effect of recycled aggregates on splitting tensile strength is ambiguous. According to some sources, splitting tensile strength may increase [39,40,41], while others claim a decrease in this index [42,43]. The workability of concrete mixtures based on recycling aggregates tends to decrease according to [44,45].

The authors note the existence of a certain gap in knowledge and the lack of a regulatory framework for the design of compositions of recycling fine-grained concrete with a maximum size of no more than 2 mm.

### 1.3. Alkali Activation Method for Recycling Concrete Properties’ Improvement

Based on the analysis of Section 1.1 and Section 1.2, it can be concluded that the use of BP and recycled aggregates is promising; however, without certain modifications of the concrete composition, it is not always possible to achieve an increase in its mechanical characteristics due to the structural features of the waste materials used. This limits the maximum content of recycled materials in concrete compositions. To enhance the interaction between components in concrete composites and improve their specified properties, it is advisable to utilize alkaline activation (AA) methods. Basic materials acting as alkaline agents can be sodium sulphate (Na_2_SO_4_), potassium sulphate (K_2_SO_4_), sodium hydroxide (NaOH), sodium silicate (Na_2_SiO_3_), sodium carbonate (Na_2_CO_3_), etc. [46,47,48,49,50].

The authors of [51] report that by optimizing cement–binder systems with active mineral admixtures and recycled aggregate, it is possible to achieve a concrete strength of 50 MPa after 28 days of hardening. The combination of AA with man-made pozzolanic materials significantly enhances the properties of modified concrete with 70% recycled aggregates [52]. This provides a concrete compressive strength of at least 60 MPa, flexural strength of at least 4.9 MPa, and splitting tensile strength of at least 3.7 MPa. Alkaline activation of hybrid recycled concrete allows for the utilization of 80–90% recycled aggregates with a compressive strength of 47 MPa [53]. The compressive strength of recycled concrete with AA and pozzolanic admixture was comparable to that of control concrete based on slag cement, ranging from 30 to 45 MPa depending on the composition [54]. Combining AA in certain proportions makes it possible to increase the strength of recycled composites by 2.5–3 times [55]. In addition, studies emphasize the positive effect of AA technology on creating new types of cement with the addition of BP [56,57,58,59,60]. This technological technique significantly reduces CO_2_ emissions in the concrete industry and can further reduce the cost of finished products [61,62]. Also worth mentioning are new research approaches using artificial intelligence to evaluate the effectiveness of alkaline activation procedures on the change of the mechanical properties of concretes and to estimate the amount of CO_2_ emissions [63,64].

### 1.4. Methods of Optimal Experiment Planning for Multifactor Composite Systems

The data mentioned above demonstrate the versatility of concrete compositions, which can be achieved through the use of different modifications such as BP, recycled aggregate, and AA. However, the majority of the mentioned studies are from the point of view of studying the complex influence of factors on the properties of the investigated concretes. This fact limits the idea of the effectiveness of the complex influence of factors in the composition of concrete on the change of its properties and parameters.

To solve this problem, it is advisable to apply methods of experimental–statistical modeling [65,66]. This allows for the minimization of the number of full-scale experiments in multifactor systems (input parameter *X_n_*—amount of cement, recycled aggregate, AA, etc.) to determine their output characteristics (*Y_n_*—strength, workability, air content, etc.) [67,68]. Based on the data obtained, it becomes possible to construct experimental models (ES-Models) that accurately depict the simultaneous influence of varying factors on the properties of concrete composites. These models are utilized to optimize the compositions for manufacturability, material consumption, and efficiency in a particular engineering task [69,70,71,72,73,74,75,76,77,78].

### 1.5. Textile-Reinforced Concrete (TRC) and Its Production Methods

Advances in science and technology are changing the technological approaches to the design and manufacture of building structures. This makes it possible to obtain stronger, more stable, and durable structures, to which TRC belongs [79,80,81]. This composite is similar to steel-reinforced concrete in its mechanical principle of performance under load. However, its main feature is the replacement of heavy steel reinforcement with light reinforcing strands made of carbon, basalt, or glass. This allows for a significant reduction in the thickness of the protective layer of concrete, as the textile reinforcement used is unaffected by corrosion. In addition, these materials have increased strength characteristics compared to steel, which helps to reduce the cross-section of structural elements and, consequently, to decrease their material consumption. As a rule, the reinforcement spacing of textile elements does not exceed 1–3 cm and their diameter does not exceed 1.5 mm. For this reason, the maximum aggregate size for TRC should not exceed 4 mm. This will ensure a high quality of monolithic behavior between the textile reinforcement and the concrete matrix [82,83,84].

The traditional method of TRC production is lamination [85]. In this method, a layer of concrete is first placed into which the textile reinforcement mesh is then embedded. This process is repeated until the desired structural thickness is achieved and is used to produce simple structural elements. As this paper explores the development of concrete for precast TRC structural elements, this method of production may not be rational due to the complexity of the geometry of the elements (I-beam, T-sections, hollow elements, etc.). Additional difficulties in production may be caused by the small distance between the textile reinforcement meshes, which requires the development of high-strength concrete mixtures with high rheological properties [86,87].

From the authors’ point of view, the use of fine-grained SCC can be a solution to this problem. Additional optimization of the production process in terms of saving energy and raw material natural resources, as well as reducing CO_2_ emissions, is possible by replacing the basic concrete components (cement and quartz sand) with BP and recycled sand (RS) with additional AA to improve the properties of the investigated SCC. This literature review shows the effectiveness of the separate applications of BP, RS and AA; however, there is a significant lack of knowledge regarding the combined effect of these materials on the properties of SCC. For this reason, this study aims to determine the combined effect of varying factors (BP, RS, and AA) on the performance of fresh and hardened SCC for the production of TRC precast elements.

## 2. Materials and Methods

**Figure 1:**
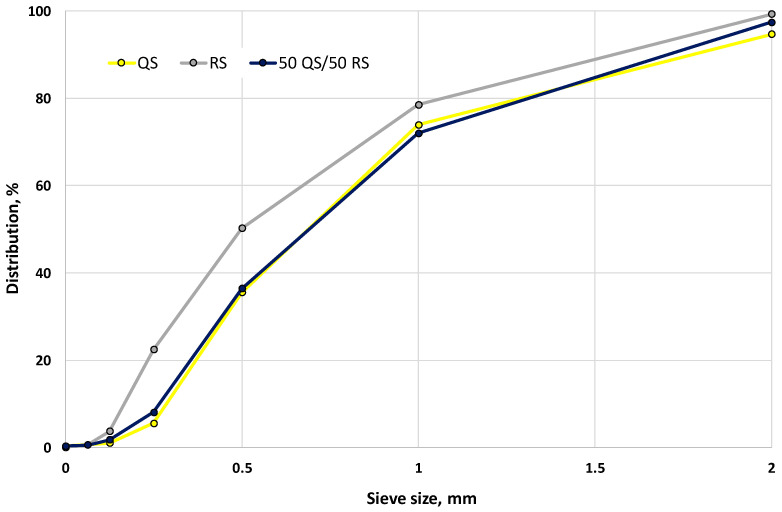
Particle size distribution of fine aggregates: QS, RS and 50% of QS with 50% of RS.

The main binder for the production of self-compacting fine-grained concrete mixtures was CEM III/A 42.2 N produced by Heidelberg Materials AG according to [88,89]. For the partial replacement of cement, 15 and 30% BP from red ceramic brick production waste of Lücking GmbH & Co. (Warburg, Germany) were used. The waste ground brick dust was used after sieving through a 63 µm sieve so that the maximum grain size of the BP was smaller than 63 µm. The fine aggregate was quartz sand (QS) from the Rhine river of fraction 0/2 [90] and recycling sand (RS) of fraction 0/2 from Heidelberg Materials AG (Heidelberg, Germany). In Figure 1, the particle size distribution of QS, RS, and mixed composition consisting of 50% QS and 50% RS are presented. The bulk density of QS was 1548 kg/m^3^, and water absorption was 1.26%; RS—1288 kg/m^3^, water absorption 1.76%; the mixed composition of 50% QS and 50% RS—1438 kg/m^3^, water absorption 1.38%. The filler was SH Compact material based on natural calcium carbonate, manufactured by SH Minerals GmbH (Heidenheim an der Brenz, Germany). Polycarboxylate superplasticizer (SP) MC-Power Flow 1102 [91] from MC-Bauchemie GmbH & Co. (Bottrop, Germany) was used to achieve the design workability of SCC mixtures. Anhydrous sodium sulphate (Na_2_SO_4_) in a quantity of 0–1% of the cement content, manufactured by Cordenka GmbH & Co. (Erlenbach am Main, Germany), was used as AA. Tap water [92] was used for blending the SCC mixtures.

The experimental–statistical modeling technique was used to study the fresh and hardened properties of the investigated SCC. A three-factor experiment based on an optimal fifteen-point symmetric plan was conducted. The following were selected as varying factors:

X_1_—amount of BP (from 0 to 234.6 kg/m^3^);

X_2_—amount of RS (from 0 to 1058 kg/m^3^);

X_3_—amount of AA (from 0 to 7.82 kg/m^3^).

Table 1 shows the compositions of the studied modified SCC using coded values of the varied factors (−1—lower level, 0—average level, and +1—upper level of variation). Different consumption of SP depended on the composition of the studied SCC to achieve a slump flow (SF) in the range of 870–880 mm, with a constant W/C ratio of 0.27. The smaller quantity of RS compared to QS per 1 m^3^ of SCC is explained by the lower density of the RS.

To achieve high quality of the investigated SCC, its preparation was carried out according to the following technological scheme:

- Preparation of sodium sulfate solution with 40% or 80% of mixing water (for the amount of AA 3.91 kg/m^3^ and 7.82 kg/m^3^, respectively). This consumption of water was chosen based on the fact that anhydrous sodium sulphate was used in the experiment, which has a water solubility of about 20%;
- Mixing of the required quantity of SP with the remaining amount of mixing water (60% or 20%) when modifying the mixture with AA, or pre-mixing of SP with 100% of mixing water for compositions without AA;
- Homogenization of cement, fine aggregate, filler, and BP for two minutes;
- Simultaneous mixing of dry mixed components with alkaline solution and water solution with SP;
- Fresh concrete mixing for 10–15 min depending on the composition.

Each batch of manufactured concrete specimens was cured: during the first 24 h of curing, while still in the formwork, a polyethylene film was used; after 24 h, the concrete was demolded and placed in a normal curing chamber (t = 20 ± 2 °C, relative humidity 65 ± 5%) until the day of testing [93].

It should be noted that, according to the requirements of the standards [94,95,96], concrete mixtures can only be classified as SCC if the following parameters are met: filling ability, passing ability, and segregation resistance. Special attention should also be paid to air entrainment, which is recommended to be maintained within 2–5%, which can be explained by the optimal relationship between the strength and durability of concrete [97,98,99].

### 2.1. Workability of SCC Mixtures (Slump Flow Test)

**Figure 2:**
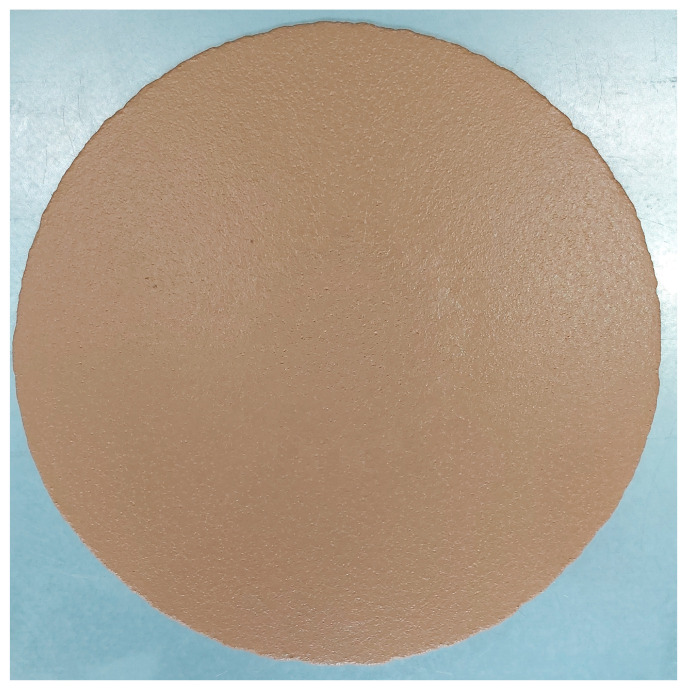
Flowability of SCC mixture No. 14 (SF = 878 mm).

The key factor determining the workability of SCC mixtures is their ability to compact under their own weight without vibration. For this purpose, the method in [100] was used to determine the flowability of the tested SCC. A standard cone was filled in one approach with the prepared SCC mixture without compaction. The cone was slowly lifted upwards in a single movement, after which the time to reach the spreading diameter t500, then the largest spreading diameter *d*_1_ and the spreading diameter *d*_2_ perpendicular to it were recorded. The final slump flow (SF) diameter was defined as the average value between *d*_1_ and *d*_2_. Figure 2 shows the result of the SF of mixture No. 14. The obtained data on the SF of the investigated SCC mixtures are given in Table 2.

### 2.2. Passing Ability of SCC Mixtures (J-Ring Test)

**Figure 3:**
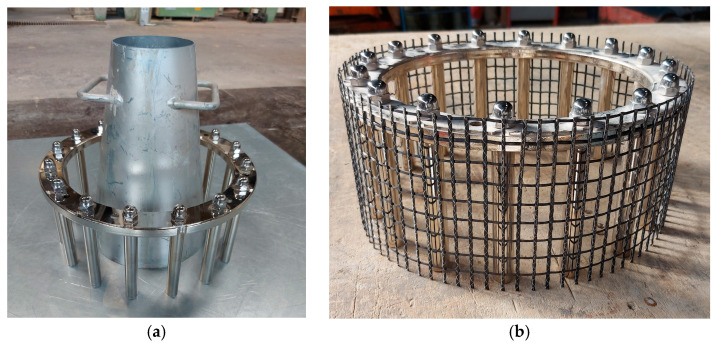
J-ring testing equipment: standard J-ring [103] (**a**), modified J-ring with carbon textile mesh (**b**).

Since the main area of application of SCC is densely reinforced structures, which includes textile-reinforced concrete [87,101,102], the studied SCC mixtures were tested for passing ability [103]. It should be noted that in addition to the standard test with J-ring (Figure 3a), similar tests were carried out with modification of the J-ring with carbon textile mesh (Figure 3b). This methodology helps to approximate the studied SCC to the real conditions of concreting of TRC elements.

**Figure 4:**
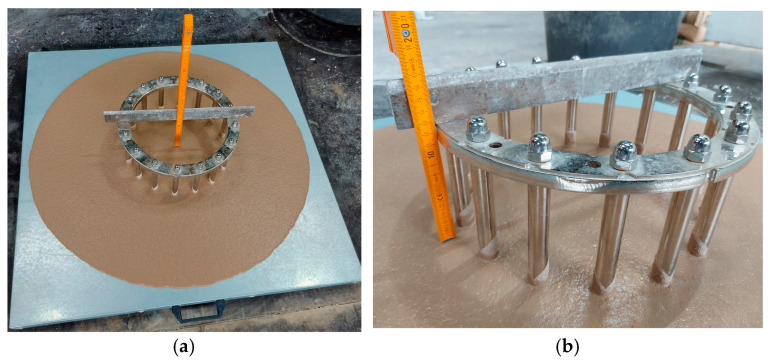
PJ test of SCC mixture № 12 (SFj-ring = 868 mm): midpoint measurment (**a**), measurement at one of the characteristic points (**b**)

To determine the passing ability (PJ), the height difference between the concrete mixture and the top of the J-ring (in the middle and two perpendicular directions—Figure 4a,b) was determined. In addition, the SF and time t500 were recorded (Table 2). Based on the data obtained, the PJ of the investigated SCC was determined, and the results are presented in Table 3.

### 2.3. Working Life of SCC (Open Time)

The open time of the SCC mixtures during time determines the possibility of production volumes of concreting considering technological pauses and changes in the conditions of the work (for example, in the hot season), and allows researchers to take into account the features of rheological properties of modified concrete mixtures with rapid strength gain with the use of AA.

The authors conducted SF tests on the investigated SCC using a standard cone, standard J-ring, and modified J-ring (with carbon textile reinforcement) at 15 and 30 min after the concrete mixtures were prepared. The results are summarized in Table 2.

### 2.4. Segregation Resistance Test

The resistance of the SCC mixtures to segregation is an important quality parameter for assessing the water bleeding of mixtures, as well as the segregation of mortar and fine aggregate. The segregation resistance of the studied SCC was determined according to the method of the standard [104], but in our case, a finer sieve (2 mm) was used as the maximum aggregate size of the examined mixtures was 2 mm. After mixing, the temperature of the concrete mixture was determined. Further, given the peculiarities of preserving the open time of the investigated SCC mixtures, they were kept for 7 min in the concrete mixer under a covered film to avoid evaporation of moisture. Then, visual assessment of the mixture condition was carried out for water bleeding. After that, the mixture was filled into a prepared sieve with a container and allowed to stand for 2 min. As a result, the mass of the concrete mixture passed through the sieve was recorded and segregation resistance (SR) was determined. The results obtained are summarized in Table 3.

### 2.5. Fresh Density and Air Content Test

The density values of the fresh concrete mixture enable the prediction of the pressure on the formwork during the concreting of structures [105]. The test was carried out according to the standard [106]. The air content (AC) of SCC is an important parameter that determines the quality of hardened concrete over time. By 1% of air content in fresh mixture, the drop in the compressive and flexural strength of hardened concrete can be 3–5% [97,98,99]. The AC test was carried out according to the pressure method [107]. The results obtained are shown in Table 3.

### 2.6. Hardened Properties of SCC

The following properties of hardened concrete were determined in this study: compressive and flexural tensile strength at the age of 1, 3, 7, and 28 days, and splitting tensile strength at the age of 28 days.

### 2.7. Compressive and Flexural Strength (CS, FS)

Compressive strength and flexural strength are the most important characteristics of concrete, reflecting its bearing capacity under the action of external load. For each batch, three prisms with dimensions 4 × 4 × 16 cm were produced. Firstly, three-point bending tests were carried out according to the method in [88]. After that, the remaining half prisms of six pieces were used for compressive strength tests [88]. The results obtained are summarized in Table 4.

### 2.8. Splitting Tensile Strength (STS)

**Figure 5:**
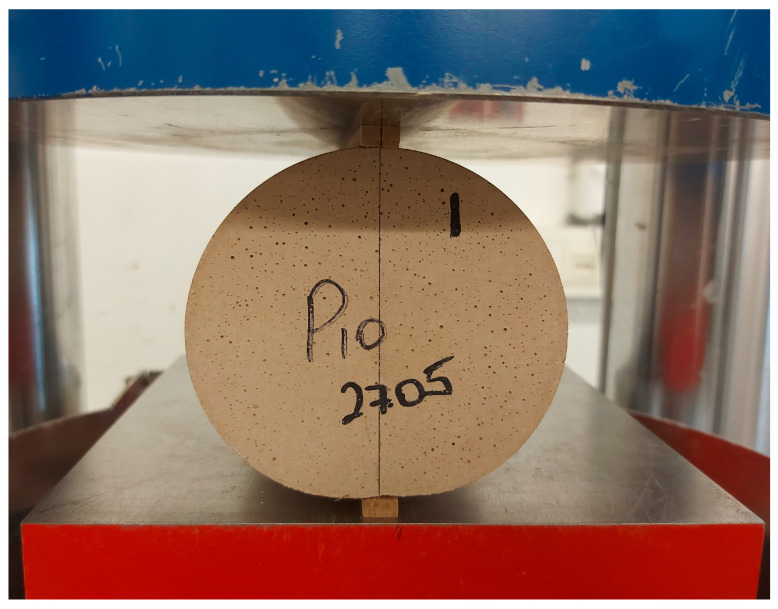
Splitting tensile strength test (mixture No. 10).

The splitting tensile strength (STS) provides a more complete picture of the behavior of concrete in flexural elements under tensile forces. This characteristic can be taken into account when predicting the properties of beams under shear force [108,109]. To determine this characteristic, three cylinders with a diameter of 100 mm and a height of 200 mm were manufactured for each batch. After 28 days of curing, tests were carried out according to the standard [110], as shown in Figure 5. The data obtained are summarized in Table 4.

## 3. Data Processing and Modeling

For the calculation and statistical analysis of ES-Models of the influence of various composition factors on the SCC properties, the COMPEX dialog system was used. This system was developed at the Odessa State Academy of Civil Engineering and Architecture under the leadership of prof. Voznesenskiy [111].

The coefficients of the ES-Model were calculated considering the accepted experimental error at 10% two-sided risk, i.e., at α = 0.1. For a given level of risk, after each calculation, a test of the hypothesis regarding whether the difference in ES-Model coefficient differs from zero was carried out, i.e., the significance of the coefficients was checked. To test the hypothesis that the calculated coefficients *b_i_* are equal to zero, the Gaussian accuracy criterion was used. The coefficients that did not differ from zero, i.e., were not significant, were consistently excluded from the ES-Model. After excluding the insignificant coefficients, the model was automatically re-calculated and the test was repeated. After the sequential analysis, the ES-Model with all significant coefficient estimates was checked for adequacy by the Fisher criterion. If the Fisher criterion was lower than the critical value for a given risk level, considering the number of degrees of freedom obtained, i.e., F_a_ < F_cr_(σ, f_df_, f_e_), the ES-Model was considered adequate, i.e., accepted for analysis and engineering decision-making. When writing the polynomials of multifactorial ES-Models, a coefficient equal to zero was written in place of the reduced insignificant elements, i.e., the value ±0 was indicated.

## 4. Experimental Results and Analysis

Table 2 shows the obtained results of the SF and open time tests of the fresh SCC mixtures. The main objective was to achieve SF in the range of 870–880 mm and to maintain this value with the J-ring and J-ring with textile reinforcement test from 850 to 860 mm. Based on this, further investigations were carried out on the open time of the SCC mixtures, SR, AC, and determination of the density of fresh concrete.

### 4.1. Slump Flow, J-Ring Test and Working Life of SCC

**Figure 6:**
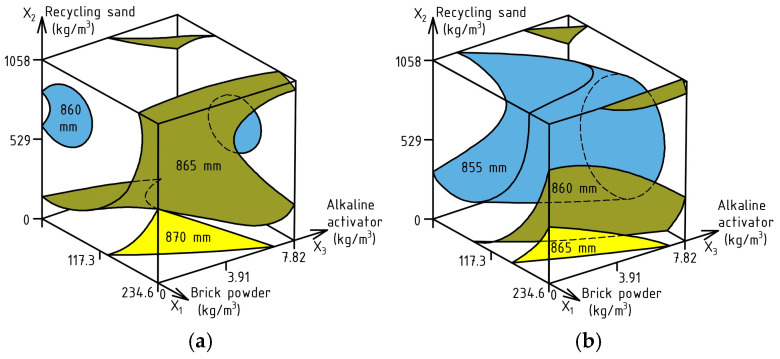
Combined effect of SCC composition factors on flowability: SF_J-ring_ (**a**), SF_J-ring textile_ (**b**).

ES-Models (1) and (2) describing the combined effect of varying factors on the flowability of SF_J-ring_ and SF_J-ring textile_ mixtures were calculated from the data in Table 2. The experimental error of model (1) was 2.37 mm and that of model (2) 2.98 mm. Using the models, the corresponding response surfaces were generated (Figure 6).

SF_J-ring_ (mm) = 860.51 + 2.3X_1_ − 1.9X_2_ − 1.1X_3_ + 3.21X_1_^2^ + 4.21X_2_^2^ ± 0X_3_^2^ − 1.5X_1_X_2_ − 2.25X_1_X_3_ + 2.5X_2_X_3_ (1)

SF_J-ring textile_ (mm) = 852.43 + 3.0X_1_ − 2.5X_2_ ± 0X_3_ + 5.43X_1_^2^ + 3.93X_2_^2^ ± 0X_3_^2^ − 2.0X_1_X_2_ − 2.0X_1_X_3_ + 3.0X_2_X_3_ (2)

Based on the data in Figure 6, we can see a slight decrease in the flowability of the investigated SCC mixtures, which did not exceed 1.9% with SF_J-ring_ and 2.9% with SF_J-ring, textile_ compared to the SF test. In turn, the difference between the flowability results with SF_J-ring_ and SF_J-ring, textile_ did not exceed 1.3%.

The diagrams show that the greatest effect on the reduction in flowability of mixtures is an increase in the content of RS (X_2_) compared to QS. Thus, by using RS in the volume of 635–845 kg/m^3^, a decrease in flowability of about 10–12 mm is observed. This is explained by the higher water absorption of this material compared to QS. However, this negative effect can be levelled by the introduction of BP (X_1_) instead of cement in the quantity of 160–175 kg/m^3^ in combination with the addition of AA (X_3_) in the amount of 1.6–2.3 kg/m^3^. It is worth considering the fact that increasing the concentration of AA solutions leads to a decrease in the length of the plasticizer chain and reduces its efficiency [112,113,114], but in our case, this is compensated by increasing the amount of SP with each increase in the amount of AA, which can be seen in Table 1.

**Figure 7:**
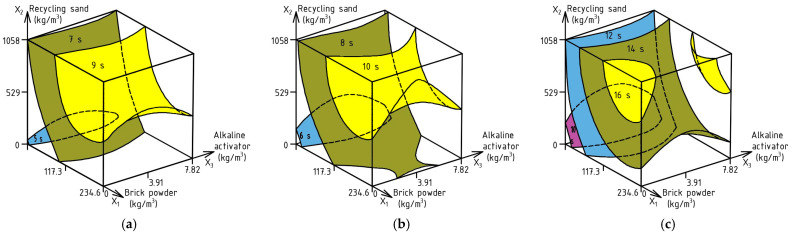
Combined effect of SCC composition factors on: SF t500 (**a**), SF t500_J-ring_ (**b**), SF t500_J-ring textile_ (**c**).

The spreading time t500 of SCC is also an important property that determines the volume of concreting per unit of time and can contribute to the acceleration of the concrete production process. To evaluate this parameter, ES-Models (3)–(5) were calculated describing the effect of factors on the t500 rate for SF, SF_J-ring_, and SF_J-ring, textile_, respectively. The experimental error was 0.47 s, 0.52 s and 0.74 s for ES-Models (3)–(5), respectively. Based on these models, the corresponding response surfaces are plotted (Figure 7a–c).

SF_t500_ (s) = 8.36 + 1.44X_1_ + 1.09X_2_ ± 0X_3_ − 1.0X_1_^2^ − 0.45X_2_^2^ + 0.45X_3_^2^ ± 0X_1_X0_2_ ± 0X_1_X_3_ ± 0X_2_X_3_ (3)

SF_t500_, _J-ring_ (s) = 9.04 + 1.53X_1_ − 1.11X_2_ ± 0X_3_ − 1.13X_1_^2^ − 0.53X_2_^2^ + 0.72X_3_^2^ ± 0X_1_X_2_ ± 0X_1_X_3_ ± 0X_2_X_3_ (4)

SF_t500_, _J-ring textile_ (s) = 14.1 + 2.28X_1_ + 1.59X_2_ ± 0X_3_ − 1.7X_1_^2^ − 0.75X_2_^2^ + 1.05X_3_^2^ ± 0X_1_X_2_ ± 0X_1_X_3_ ± 0X_2_X_3_ (5)

Data analysis of the surfaces in Figure 7 shows the predicted reduction in the t500 rate of the investigated SCC depending on the test method. Therefore, the decrease between SF t500 and SF t500_J-ring_ is 5.6–15.3% depending on the composition of the SCC, and between SF t500 and SF t500_J-ring textile_, it reaches 65.1–85.7%. In turn, the difference in the SCC flow rate between SF t500_J-ring_ and SF t500_J-ring textile_ is in the range of 53.6–66.7%.

The greatest influence on the decrease in the flow time of SCC is the increase in the amount of BP and RS. This is due to the reduction in the amount of liquid phase with a declining mass of cement, and the rough surface of RS prevents the increase in the spreading time of the mixture. To ensure the speed of the SF t500_J-ring textile_ within 8–10 s, it is possible to partially replace cement with BP in the range of 90–115 kg/m^3^, as well as the use of RS in the volume of 370–475 kg/m^3^ and the addition of AA in the amount of 3.2–5.1 kg/m^3^. With the same compositional modifications, the SF t500 rate will be within 5–6 s and SF t500_J-ring_ 6–7 s.

### 4.2. Working Life of SCC

To determine the effect of varying composition factors on the open time of SCC, it is not possible to calculate adequate ES-Models due to the lack of a dataset due to the loss of SCC properties of the studied mixtures, which are presented in Table 2. Nevertheless, analyzing the data in Table 2 allows us to conclude that there is a negative effect of increasing the quantity of each of the varying factors on the retention of the SF of the SCC over 15 and 30 min. To maintain the specified SF and spreading time properties of SCC, the amount of AA should not exceed 3.91 kg/m^3^. At the same time, it is possible to substitute cement with BP in the amount of 117.3 kg/m^3^ in combination with the replacement of QS with RS in the volume of 1058 kg/m^3^ or it is possible to increase the consumption of BP content to 234.6 kg/m^3^ with using RS in the volume of 529 kg/m^3^.

### 4.3. Passing Ability, Segregation Resistance, Air Content, Fresh Density, Temperature and Mixing Time of SCC Mixtures

Table 3 summarizes the results of experimental studies of the studied SCC: passing ability (PJ), segregation resistance (SR), air content (AC), fresh density, temperature, and mixing time.

The temperature of fresh concrete mixtures did not exceed 25 °C, which is satisfactory. The mixing time of the mixtures to achieve the required rheological performance was in the range of 10–15 min depending on the mixture composition. It should be noted that as the volume of recycled materials increased, a decrease in the density of fresh SCC was observed.

**Figure 8:**
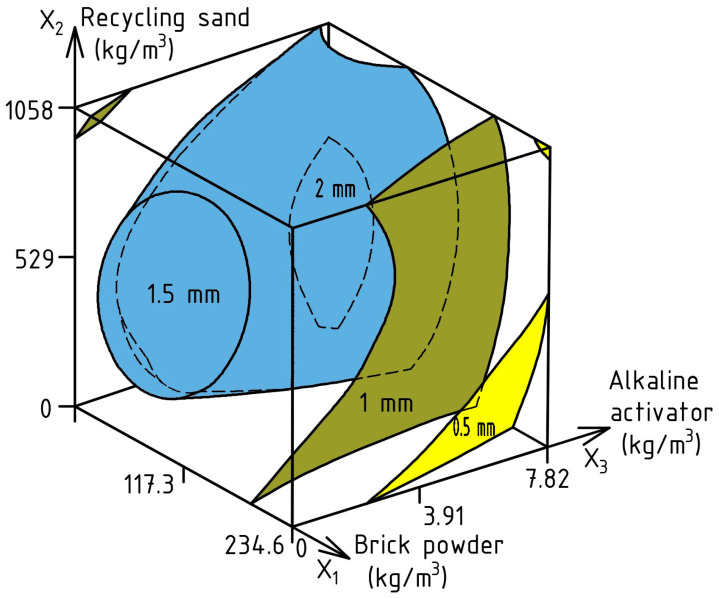
Combined effect of SCC composition factors on PJ.

Based on the data in Table 3, ES-Model (6) was calculated for PJ, the error of the experiment was 0.31 mm, and the obtained response surface is shown in Figure 8.

PJ (mm) = 1.65 − 0.41X_1_ ± 0X_2_ ± 0X_3_ − 0.38X_1_^2^ − 0.28X_2_^2^ ± 0X_3_^2^ + 0.25X_1_X_2_ − 0.38X_1_X_3_ ± 0X_2_X_3_ (6)

The data in Figure 8 show that increasing the amount of BP instead of cement can reduce the PJ value by 2–4 times to values of 1–0.5 mm. This is especially important for densely reinforced structures with textile reinforcement. The positive effect of RS in the volume of 480–610 kg/m^3^ on PJ reduction should also be emphasized. With increasing the AA dosage, the PJ did not exceed 2 mm, which meets the requirements [94,95,96].

**Figure 9:**
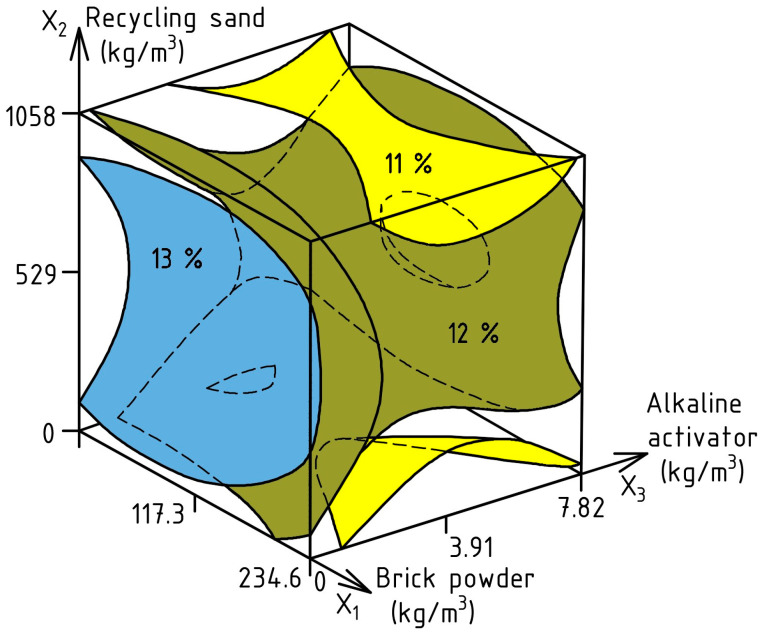
The influence of SCC composition factors on SR.

To evaluate the effect of experimental factors on SR, ES-Model (7) was calculated; the response surface is shown in Figure 9, and the experimental error was 0.49%.

SR (%) = 12.32 − 0.29X_1_ ± 0X_2_ − 0.48X_3_ − 0.52X_1_^2^ − 1.36X_2_^2^ + 1.23X_3_^2^ + 0.23X_1_X_2_ ± 0X_1_X_3_ ± 0X_2_X_3_ (7)

From the data diagram, it can be concluded that partial replacement with BP slightly reduces the SR of mixtures in the range of 2–3%. In turn, the use of RS in the volume of 680–1058 kg/m^3^ achieves an SR of about 12%, and modification with AA in the range of 3.12–5.8 kg/m^3^ can also reduce the SR to 11%. Considering that fine-grained mixtures are used in this study, SR plays a major role in ensuring the quality of SCC. Accordingly, at the optimum dosage of varying composition factors, this indicator can be in the range of 11–13%, which corresponds to the requirements in [94,95,96].

**Figure 10:**
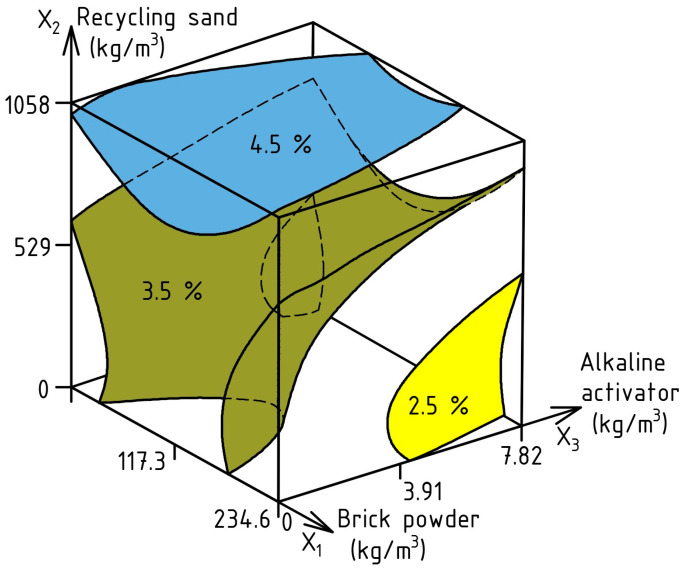
Effect of varying composition factors of SCC on AC.

The AC of the investigated SCC was analyzed based on the calculated ES-Model (8); the response surface is shown in Figure 10, and the experiment error was 0.35%.

AC (%) = 8.8 − 0.21X_1_ ± 0X_2_ − 0.34X_3_ − 0.37X_1_^2^ − 0.97X_2_^2^ + 0.88X_3_^2^ + 0.16X_1_X_2_ ± 0X_1_X_3_ ± 0X_2_X_3_ (8)

The data in Figure 10 show the effectiveness of partial substitution of cement with BP to reduce the volume of entrained air. Consequently, with an amount of BP in the range of 165–230 kg/m^3^, the AC of SCC can be in the range of 3–4.4%. Increasing the quantity of AA also contributes to the reduction in AC, which, at a maximum consumption of 7.8 kg/m^3^, does not exceed 4.5%. The optimum quantity of RS is 210–625 kg/m^3^, at which the AC of the mixture was 3.7–4.4%. Increasing the volume of RS above 625 kg/m^3^ is not rational due to the sharp increase in the amount of air in the fresh concrete.

### 4.4. Compressive, Flexural and Tensile Splitting Strength

**Figure 11:**
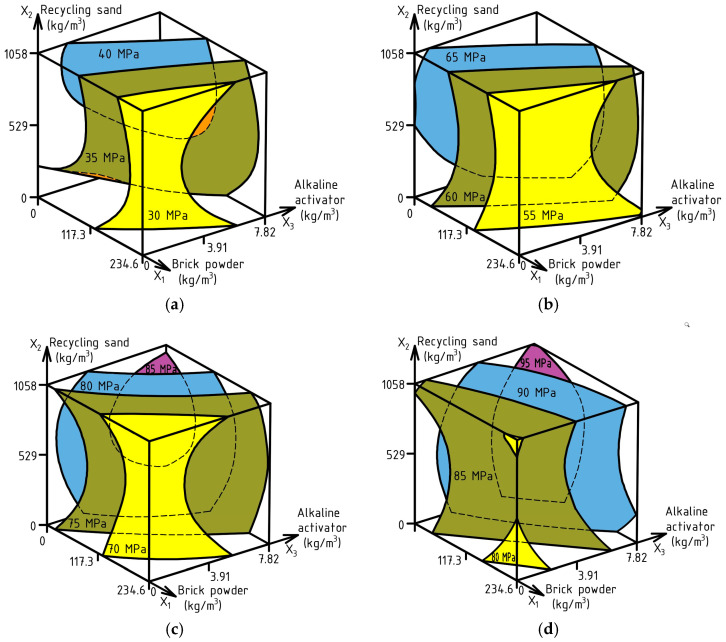
Combined effect of SCC composition factors on: CS_1_ (**a**), CS_3_ (**b**), CS_7_ (**c**), CS_28_ (**d**).

Based on the experimental data from Table 4, ES-Models (9)–(12) were calculated for CS at ages of 1, 3, 7, and 28 days, with corresponding experimental errors of 1.48 MPa, 1.38 MPa, 1.71 MPa, and 2.69 MPa. The response surfaces are reflected in Figure 11.

CS_1_ (MPa) = 37.18 − 4.78X_1_ + 1.71X_2_ + 2.81X_3_ − 1.39X_1_^2^ − 2.47X_2_^2^ ± 0X_3_^2^ − 1.64X_1_X_2_ ± 0X_1_X_3_ ± 0X_2_X_3_ (9)

CS_3_ (MPa) = 63.02 − 6.83X_1_ + 1.07X_2_ + 3.22X_3_ − 1.64X_1_^2^ − 2.23X_2_^2^ ± 0X_3_^2^ ± 0X_1_X_2_ ± 0X_1_X_3_ ± 0X_2_X_3_ (10)

CS_7_ (MPa) = 78.89 − 6.35X_1_ ± 0X_2_ + 4.39X_3_ − 1.46X_1_^2^ − 3.08X_2_^2^ − 1.74X_3_^2^ ± 0X_1_X_2_ ± 0X_1_X_3_ ± 0X_2_X_3_ (11)

CS_28_ (MPa) = 89.31 − 3.69X_1_ ± 0X_2_ + 5.64X_3_ ± 0X_1_^2^ − 1.97X_2_^2^ ± 0X_3_^2^ + 1.35X_1_X_2_ ± 0X_1_X_3_ ± 0X_2_X_3_ (12)

As can be seen from the diagram in Figure 11a,b, the obtained SCC can be classified as fast-hardening. The early CS at the age of 1 day was in the range of 21–44 MPa (28–47% of the design strength of concrete), and at the age of 3 days, it already ranged from 47 to 71 MPa (58–76% of the design CS) depending on the composition. So, at the partial substitution of cement with BP in the amount of 110–130 kg/m^3^ in combination with replacement of NS with RS in the volume of 540–635 kg/m^3^ and addition of AA in the amount of 1.6 kg/m^3^, it is possible to obtain SCC with CS on the first day of hardening at 40 MPa, and to obtain concrete with CS not lower than 60 MPa on the third day of hardening. Indicators of early strength are especially important in the production of precast elements of reinforced structures, in particular, TRC. High early strength increases formwork turnover and production volumes. Without achieving high early strength indicators, it is also impossible to move large reinforced concrete elements for storage.

It is necessary to notice that the increase in the partial substitution of cement with BP from 15 to 30% has a more pronounced effect of reducing early strength than in the case of partial replacement from 0 to 15%. In turn, this is compensated by increasing the dosage of AA, while the use of more than 75% of RS is not rational from the point of view of the effect of this factor on the CS at the age of 1 and 3 days. A similar trend is observed for CS at the age of 7 and 28 days (Figure 11c,d). Increasing the amount of AA to 5.45 kg/m^3^ in combination with cement replacement with BP in the quantity of 130 kg/m^3^ in combination with RS in the volume of 635 kg/m^3^ allows us to obtain concrete with CS at 7 days of not less than 72 MPa, and at the age of 28 days of not less than 90 MPa, which refers to the class of concrete C60/70 [115].

**Figure 12:**
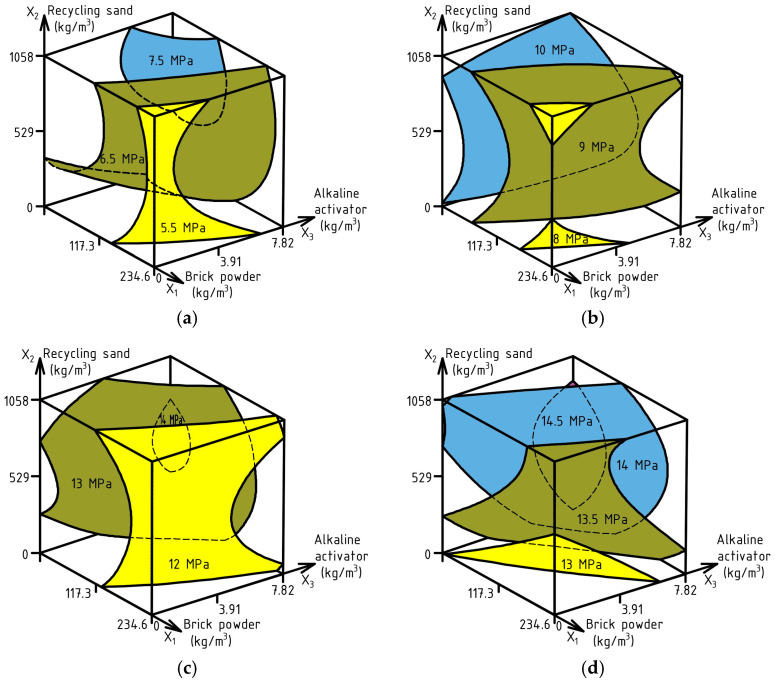
Compound effect of SCC composition factors on: FS_1_ (**a**), FS_3_ (**b**), FS_7_ (**c**), FS_28_ (**d**).

ES-Models (13)–(16) with experimental errors of 0.3 MPa, 0.37 MPa, 0.35 MPa, 0.22 MPa were calculated to analyze the influence of the factors on FS at ages of 1, 3, 7, and 28 days. The response surfaces are shown in Figure 12.

FS_1_ (MPa) = 6.94 − 0.71X_1_ + 0.38X_2_ + 0.48X_3_ − 0.42X_1_^2^ − 0.45X_2_^2^ ± 0X_3_^2^ − 0.18X_1_X_2_ ± 0X_1_X_3_ ± 0X_2_X_3_ (13)

FS_3_ (MPa) = 9.73 − 0.97X_1_ ± 0X_2_ + 0.43X_3_ ± 0X_1_^2^ − 0.74X_2_^2^ ± 0X_3_^2^ + 0.19X_1_X_2_ + 0.18X_1_X_3_ ± 0X_2_X_3_ (14)

FS_7_ (MPa) = 13.06 − 0.87X_1_ ± 0X_2_ + 0.5X_3_ − 0.3X_1_^2^ − 0.63X_2_^2^ ±0X_3_^2^ ± 0X_1_X_2_ ± 0X_1_X_3_ ± 0X_2_X_3_ (15)

FS_28_ (MPa) = 13.85 − 0.47X_1_ + 0.32X_2_ + 0.45X_3_ ± 0X_1_^2^ − 0.37X_2_^2^ ± 0X_3_^2^ + 0.16X_1_X_2_ ± 0X_1_X_3_ − 0.3X_2_X_3_ (16)

The FS after 1 day of hardening was 5.1–8 MPa (37–53% of the FS strength at the age of 28 days), and after 3 days of hardening, it was from 7.8 to 11.5 MPa (56–77% of the design flexural strength at the age of 28 days) depending on the composition of the examined SCC. The use of BP in the amount of 105–120 kg/m^3^ to replace cement with the combined application of RS in the quantity of 750–960 kg/m^3^ and the inclusion of 6.25 kg/m^3^ AA allows us to obtain concrete with FS at 1 day of 7.5 MPa, and at 3 days of not less than 10 MPa. It is important to emphasize that increasing the quantity of AA up to 1% of the cement content increases the FS on the first day, and the use of AA in the amount of 0.9–1% of the cement content does not increase this strength index at 3 days of hardening. Accordingly, the use of more than 960 kg/m^3^ of RS does not lead to an increase in FS at the age of 1 day, and neither does the use of more than 590 kg/m^3^ at the age of 3 days. At 7 days of hardening, the same tendency of RS influence on FS remains, and increasing the quantity of AA increases this index. On the 28th day, the rational volume of RS is 420–580 kg/m^3^ in combination with AA up to 1% of binder mass. It needs to be noted that the efficiency of BP without the use of AA sharply decreases in terms of flexural strength at early and design ages.

**Figure 13:**
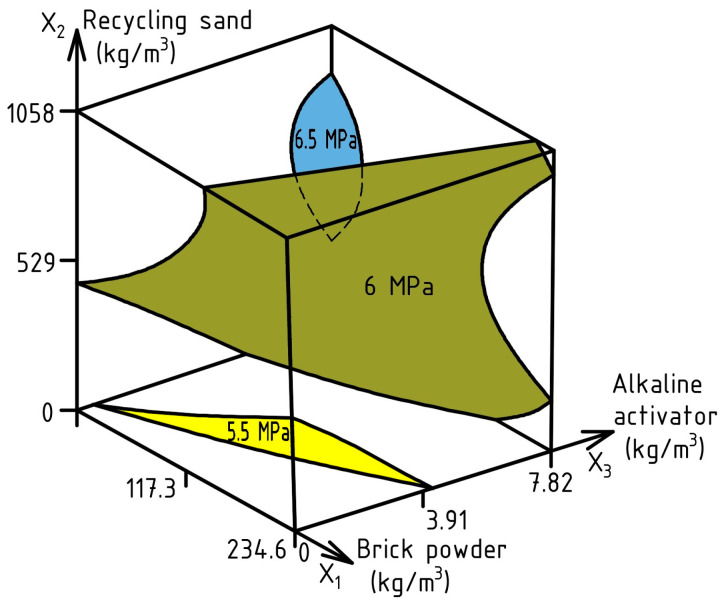
Simultaneous effect of SCC composition factors on STS.

For STS at the age of 28 days, ES-Model (17) was calculated with an experimental error of 0.18 MPa. The corresponding response surface is presented in Figure 13.

STS_28_ (MPa) = 6.07 − 0.23X_1_ + 0.24X_2_ + 0.25X_3_ ± 0X_1_^2^ − 0.19X_2_^2^ ± 0X_3_^2^ ± 0X_1_X_2_ ± 0X_1_X_3_ − 0.18X_2_X_3_ (17)

As can be seen from the diagram, increasing RS and AA to the volumes of 690 kg/m^3^ and 7.82 kg/m^3^, respectively, has the greatest effect on increasing STS. Meanwhile, increasing the amount of BP to a maximum number of 234.6 kg/m^3^ of cement can reduce STS index by no more than 6.5%. Since STS characterizes the behavior of concrete under tensile stresses, the effect of varying factors on this characteristic is similar to their effect on FS. Based on this, to achieve an STS of 6 MPa, there is a wide range of combinations of varying factors. This makes it possible to reduce the amount of cement to 20–25% and effectively use 50 to 80% RS instead of NS, which has a positive effect on the resistance of flexural elements in inclined sections under shear force [108,109].

## 5. Conclusions

The studies carried out using a three-factor optimal 15-point plan make it possible to investigate the effect of varying factors on the properties of fresh and hardened SCC for the production of textile-reinforced concrete. This contributes to the possibility of optimizing SCC compositions by selecting the required quantity of brick powder to substitute cement binder and recycling sand to quartz sand by using an alkaline activator. The main conclusions of this study are as follows:

- For an efficient use of an alkaline activator, the need to increase the dosage of superplasticizer to achieve the design flowability should be considered, as an alkaline activator has a negative effect on the slump flow;
- The use of textile reinforcement in the J-ring test reduces the flow rate t500 by more than 50% compared to the standard methodology;
- To maintain the fresh properties of the self-compacting concrete over time (up to 30 min), it is not recommended to use alkaline activator as more than 0.5% of cement content;
- Considering the segregation risks of fine-grained self-compacting concrete, the obtained ES-Models allow for the selection of concrete compositions with segregation resistance in the range of 11–13% with the maximum permissible value of 15% [94,95,96], which preserves its quality;
- To provide a balance between open porosity and strength of the modified self-compacting concrete, the air content should be in the range of 2.5–4%;
- The use of recycling materials reduces the density of fresh and hardened concrete by up to 7%, which reduces the overall weight of concrete structures;
- The calculated ES-Models enable the design of the compositions of self-compacting concrete compressive strength classes C45/55, C50/60, C55/67 with high early compressive strength not lower than 30 MPa at the age of 1 day.

Thus, on the basis of the experimental data obtained, it is possible to produce sustainable alkaline-activated self-compacting concrete with specified rheological and strength properties. The cement saving can reach up to 30%, and the saving of natural sand sources up to 100%.

Further research is planned to focus on the microstructural analysis and study of long-term durability properties such as capillary water absorption, pore structure, carbonation resistance, autogenous shrinkage, and drying shrinkage. Also, special attention should be paid to the study of the influence of plasticizers with different polycarboxylate groups in combination with different types of alkaline activators on the preservation of the rheological properties of self-compacting concrete.

## Figures and Tables

**Table 1:** Experimental plan and compositions of the investigated modified SCC.

No. of Mixture	X_1_, BP	X_2_, RS	X_3_, AA	Cement, kg/m^3^	QS, kg/m^3^	Filler, kg/m^3^	SP, kg/m^3^	BP (X_1_), kg/m^3^	RS (X_2_), kg/m^3^	AA (X_3_), kg/m^3^	Water, kg/m^3^	W/C
1	−1	−1	−1	782	1183	226	9.69	0	0	0	211.14	0.27
2	−1	−1	1	782	1183		9.75	0	0	7.82		
3	−1	0	0	782	591.5		10.26	0	529	3.91		
4	−1	1	−1	782	0		15.78	0	1058	0		
5	−1	1	1	782	0		18.77	0	1058	7.82		
6	0	−1	0	664.7	1183		10.32	117.3	0	3.91		
7	0	0	−1	664.7	591.5		11.97	117.3	529	0		
8	0	0	0	664.7	591.5		13.42	117.3	529	3.91		
9	0	0	1	664.7	591.5		14.05	117.3	529	7.82		
10	0	1	0	664.7	0		16.98	117.3	1058	3.91		
11	1	−1	−1	547.4	1183		9.71	234.6	0	0		
12	1	−1	1	547.4	1183		10.22	234.6	0	7.82		
13	1	0	0	547.4	591.5		16.67	234.6	529	3.91		
14	1	1	−1	547.4	0		16.91	234.6	1058	0		
15	1	1	1	547.4	0		17.94	234.6	1058	7.82		

**Table 2:** SF test results combined with open time tests of SCC mixtures (‘-‘ means loss of self-compacting properties of the mixture).

No. of Mixture	SF, mm	SF t_500_, s	15 min		30 min				15 min		30 min				15 min		30 min	
			SF_15_, mm	SF_15 (t500)_, s	SF_30_, mm	SF_30 (t500)_, s	J-Ring, mm	J-Ring t_500_, s	J-Ring_15_, mm	J-Ring_15 (t500)_, s	J-Ring_30_, mm	J-Ring_30 (t500),_ s	J-Ring _textile_, mm	J-Ring _textile (t500)_, s	J-Ring _textile15_, mm	J-Ring _textile15 (t500)_, s	J-Ring _textile30_, mm	J-Ring _textile30 (t500)_, s
1	880	4.6	806	8.1	615	17.0	870	5.1	776	9.2	581	19.7	865	8.5	715	21.6	502	50.5
2	875	4.9	599	13.3	-	-	865	5.6	-	-	-	-	858	9.1	-	-	-	-
3	874	6.5	603	15.1	-	-	859	6.9	-	-	-	-	849	10.8	-	-	-	-
4	870	6.8	604	14.6	-	-	861	7.6	-	-	-	-	855	11.8	-	-	-	-
5	881	6.8	572	14.7	-	-	869	7.5	-	-	-	-	863	11.6	-	-	-	-
6	878	6.3	798	8.4	667	13.7	864	6.9	763	9.5	625	16.0	855	10.9	690	24.9	526	44.4
7	875	8.5	774	10.7	522	15.8	859	9.8	740	12.9	490	-	851	15.2	655	28.5	412	-
8	873	9.4	824	11.5	634	25.3	861	10.3	782	13.1	592	23.8	854	15.9	724	25.1	513	47.9
9	868	8.6	-	-	-	-	860	9.1	-	-	-	-	854	14.2	-	-	-	-
10	881	9.0	794	11.8	715	19.2	867	9.5	753	13.0	655	21.5	856	14.9	664	26.4	528	46.5
11	886	8.1	770	9.4	694	13.6	879	8.8	742	10.6	651	16.1	875	13.7	648	20.7	522	35.6
12	875	8.0	-	-	-	-	868	9.0	-	-	-	-	863	14.0	-	-	-	-
13	882	7.7	832	9.5	736	15.4	870	8.3	796	10.6	680	17.4	865	13.1	726	23.8	570	41.8
14	878	9.8	-	-	-	-	867	10.7	-	-	-	-	860	16.6	-	-	-	-
15	874	10.4	-	-	-	-	863	11.2	-	-	-	-	857	17.2	-	-	-	-

**Table 3:** Test results of additional properties of fresh SCC mixtures.

No. of Mixture	PJ, mm	SR, %	Fresh Density, kg/m^3^	AC, %	Temperature, °C	Mixing Time, min
1	1.5	12.3	2300	3.3	24.2	10
2	1.8	11.8	2320	2.1	24.9	12
3	2.0	12.2	2280	2.5	23.5	13
4	0.5	11.9	2240	4.7	24.7	15
5	1.5	11.8	2250	4.5	24.6	14
6	1.0	11.1	2240	3.7	22.7	12
7	2.0	14.8	2230	4.5	23.3	13
8	1.5	13.0	2250	3.5	23.8	10
9	1.5	11.9	2260	3.2	24.6	15
10	1.7	10.5	2230	4.6	24.0	14
11	0.5	11.5	2270	2.6	22.4	15
12	0.5	10.8	2260	2.3	23.1	15
13	0	11.1	2220	3.0	22.8	15
14	0.5	12.0	2210	4.2	23.7	14
15	1.7	11.6	2220	3.8	24.1	15

**Table 4:** Experimental data of CS, FS and STS of investigated SCC at 1, 3, 7 and 28 days (‘±’ denotes standard deviation).

No. of Mixture	CS_1_, MPa	CS_3_, MPa	CS_7_, MPa	CS_28_, MPa	FS_1_, MPa	FS_3_, MPa	FS_7_, MPa	FS_28_, MPa	STS_28_, MPa	Density, kg/m^3^
1	31.7 ± 0.64	60.8 ± 0.65	74.5 ± 1.12	83.5 ± 1.13	5.4 ± 0.37	9.6 ± 0.56	12.2 ± 0.17	12.8 ± 0.43	5.24± 0.33	2250
2	38.0 ± 0.52	66.5 ± 1.16	83.0 ± 1.13	95.4 ± 0.6	6.7 ± 0.47	10.2 ± 0.5	13.5 ± 0.54	14.5 ± 0.67	6.33 ± 0.51	2290
3	41.9 ± 0.77	71.2 ± 1.03	88.7 ± 0.92	99.3 ± 1.15	8.0 ± 0.16	11.5 ± 0.31	14.5 ± 0.40	14.9 ± 0.44	6.84 ± 0.44	2250
4	39.6 ± 0.32	64.5 ± 1.16	74.9 ± 1.05	83.9 ± 0.98	6.6 ± 0.16	9.6 ± 0.20	12.4 ± 0.57	13.9 ± 0.69	6.19 ± 0.69	2180
5	41.8 ± 1.06	69.2 ± 0.66	83.5 ± 1.00	94.5 ± 0.78	7.6 ± 0.14	9.8 ± 0.73	12.9 ± 0.70	14.2 ± 0.68	6.31 ± 0.55	2210
6	31.6 ± 0.32	58.9 ± 0.72	76.8 ± 1.17	89.3 ± 1.07	6.2 ± 0.19	9.0 ± 0.49	12.3 ± 0.56	13.2 ± 0.74	5.6 ± 0.24	2200
7	32.6 ± 0.40	59.1 ± 0.64	72.9 ± 1.06	82.4 ± 1.16	6.3 ± 0.20	9.2 ± 0.39	13.0 ± 0.34	13.6 ± 0.39	5.82 ± 0.59	2170
8	36.4 ± 0.44	63.4 ± 0.76	81.0 ± 1.03	90.3 ± 0.86	6.9 ± 0.66	9.7 ± 0.61	13.1 ± 0.33	13.7 ± 0.48	5.94 ± 0.23	2200
9	41.7 ± 0.44	66.8 ± 1.07	82.8 ± 1.16	94.9 ± 1.00	7.3 ± 0.45	10.3 ± 0.29	13.5 ± 0.32	14.1 ± 0.67	6.23 ± 0.33	2220
10	38.6 ± 0.72	61.5 ± 0.93	76.3 ± 0.80	86.6 ± 0.54	7.1 ± 0.33	8.8 ± 0.35	12.2 ± 0.63	13.7 ± 0.61	6.05 ± 0.57	2170
11	28.0 ± 0.48	49.8 ± 0.64	63.5 ± 1.15	75.2 ± 1.12	5.1 ± 0.29	7.8 ± 0.30	11.1 ± 0.44	11.9 ± 0.48	5.17 ± 0.42	2200
12	30.1 ± 0.37	55.1 ± 0.85	73.6 ± 0.84	89.9 ± 1.13	5.5 ± 0.26	8.2 ± 0.57	11.7 ± 0.28	13.4 ± 0.35	5.86 ± 0.47	2220
13	30.5 ± 0.39	51.3 ± 0.57	67.6 ± 1.22	79.6 ± 0.91	5.3 ± 0.30	8.0 ± 0.49	11.0 ± 0.36	12.9 ± 0.48	5.52 ± 0.39	2160
14	24.1 ± 0.46	49.4 ± 0.49	64.7 ± 1.07	84.3 ± 0.98	5.1 ± 0.21	7.6 ± 0.44	10.5 ± 0.47	13.4 ± 0.34	5.93 ± 0.68	2140
15	32.5 ± 0.51	58.1 ± 0.49	71.7 ± 1.12	90.9 ± 0.94	6.3 ± 0.13	9.4 ± 0.64	12.5 ± 0.49	13.9 ± 0.17	6.15 ± 0.36	2170

## Data Availability

The data supporting this study are available from the author on request. The data are not publicly available due to restriction privacy.
